# Overexpression of members of the microRNA-183 family is a risk factor for lung cancer: A case control study

**DOI:** 10.1186/1471-2407-11-393

**Published:** 2011-09-15

**Authors:** Wangyu Zhu, XiaoGuang Liu, JianYing He, DongDong Chen, YanYan Hunag, Yong Kui Zhang

**Affiliations:** 1 Department of Cardio-Thoracic Surgery, Zhoushan Hospital of Zhejiang Province, Zhoushan, Zhejiang, 316004, 316004, China; 2 Joint Laboratory of Immunogenomics, Zhoushan Hospital-BIG/CAS, Zhoushan, Zhejiang Province, 316004, China

**Keywords:** miRNA, diagnosis, prognosis, sera, RT-quantitative PCR, human lung cancer

## Abstract

**Background:**

Lung cancer is the leading cause of cancer-related deaths worldwide. Early detection is considered critical for lung cancer treatment. MicroRNAs (miRNAs) have shown promise as diagnostic and prognostic indicators. This study was to identify specific miRNAs with diagnostic and prognostic value for patients with lung cancer, and to explore the correlation between expression profiles of miRNAs and patient survival.

**Methods:**

Gene expression of members of the miR-183 family (miR-96, miR-182, and miR-183) were examined in 70 paired samples from lung cancer patients (primary cancer and non-cancerous tissues and sera), as well as 44 serum samples from normal volunteers and lung cancer cell lines by quantitative real-time reverse transcription polymerase chain reaction (RT-qPCR). The correlation between the expression of miRNAs in tissues, sera, and patient overall survival were also examined by log-rank and Cox regression analysis.

**Results:**

Expression levels of members of the miR-183 family in lung cancer tumor and sera were higher than that of their normal counterparts. The miR-96 expression in tumors was positively associated with its expression in sera. Log-rank and Cox regression analyses demonstrated that high expression of tumor and serum miRNAs of the miR-183 family were associated with overall poor survival in patients with lung cancer.

**Conclusions:**

Our results suggest that the expressions of miR-96, miR-182, and miR-183 in tumor and sera may be considered potential novel biomarkers for the diagnosis and prognosis of lung cancer.

## Background

Lung cancer is the leading cause of cancer-related deaths worldwide [1], and the current pathologic staging is inadequate to predict outcome for patient treatment. The development of molecular target therapy has improved the management of patients with lung cancer who are at high risk of relapse following surgery. One of the most promising classes of molecular markers in tumor prognosis is the small noncoding RNAs, or microRNAs (miRNAs) [2-4]. MiRNAs, acting as oncogenes or tumor suppressors, have been shown to regulate the expression of hundreds of targeted genes at the posttranscriptional level and are implicated in the pathogenesis and therefore prognosis of human cancers [5-9]. For non-small cell lung cancer (NSCLC) in particular, previous reports have indicated that miRNA expression patterns could be potential biomarkers used for diagnosis, prognosis, and personalized therapy [4,8-11]. In addition, some studies found that human serum or plasma contained large numbers of stable miRNAs and that the expression profiles of some specific circulating miRNAs could be useful in the diagnosis and prognosis of cancer [12-14].

In our study, we performed a miRNA expression array by quantitative real-time reverse transcription polymerase chain reaction (RT-qPCR) and identified the expression of members of the miR-183 family (miR-96, miR-182, and miR-183) when comparing primary NSCLC tumor with adjacent normal lung tissues. The miR-183 family is located on human chromosome 7 and members of this family have been identified as potential oncogenes in several tumor types, including medulloblastomas [15], breast cancer [16], prostate cancer [17], hepatocellular tumors [18], and colon cancer [19,20], as well as lung cancer [21,22]. However, the role that these miRNAs play in the diagnosis and prognosis of lung cancer patients remains unknown.

We compared the expression levels of the miR-183 family in lung cancer cell lines with normal lung cells, and in primary tumor tissues and sera from NSCLC patients with normal volunteers. Our results indicate for the first time that members of the miR-183 family expressed in tumors and sera may be potential biomarkers in the diagnosis and prognosis of human lung cancer.

## Methods

### Sample population

NSCLC and matched adjacent noncancerous tissues were collected from patients undergoing lung resection surgery from January 2008 to May 2008 at Zhoushan Hospital, Zhejiang Province, China. The details of patients were shown in Table 1. Eligible samples were obtained from primary lung cancer that had not received any preoperative radiotherapy or chemotherapy, and in which there was no co-existing disease. Moreover, we excluded tissue blocks of mixed histology, or specimens with insufficient tumor material. The subtypes included 36 squamous-cell carcinoma and 34 adenocarcinoma. All human materials were obtained with patients' informed consent and the Ethical Review Committee of Zhoushan Municipal Government of China approved this study.

**Table 1:** Clinico-pathological Characteristics of 70 Patients with NSCLC

Characteristics	n
Mean age	
< 60	34
≥60	36
**Sex**	
**Male**	**56**
**Female**	**14**
Smoking	
Nonsmokers	24
Current smokers	46
**Tumor size**	
**0-3 cm**	**18**
**> 3 cm**	**52**
Histological classification	
Adenocarcinoma	34
SCC	36
**Invasion to lung membrane**	
**Yes**	**17**
**No**	**53**
differentiation	
Mod-well	46
Poor	24
**Lymph node**	
**Negative**	**38**
**Positive**	**32**
Stage classification	
StageI	36
StageII,III and IV	34

Upon removal, the surgical specimens were immediately transported to the clinical pathology laboratory, where each sample was placed in a cryovial and flash-frozen in liquid nitrogen within 30 minutes and then stored at -80°C until analyzed. Sera from all NSCLC patients and healthy volunteers were also collected. There was no significant difference in gender or age between NSCLC patients and healthy volunteers. All cases were reviewed by two pathologists and diagnoses were confirmed according to the criteria recently established by the National Comprehensive Cancer Network (NCCN).

### Cell lines and cell culture conditions

The following cell lines were cultured individually in RPMI-1640 medium (Gibco): A549, H1299, and SPC-A1 human lung adenocarcinoma; 95C and 95D human giant-cell lung carcinoma; NCI-H466 human small cell lung carcinoma; NCI-H460 human large-cell lung carcinoma; and human bronchial epithelia. In addition, SK-MES1 human squamous-cell lung carcinoma cells were cultured in Dulbecco's modified Eagle's medium (DMEM; Gibco). Both media were supplemented with 10% fetal bovine serum (Gibco), 2 mM L-glutamine, 100 IU/mL penicillin, and 100 mg/mL streptomycin. Cells were incubated in 5% CO_2 _at 37°C.

### RNA isolation

Total RNA isolated from 6 NSCLC and 6 matched adjacent noncancerous tissues by the Trizol (Invitrogen) method were prepared for miRNA microarray according to the manufacturer's instructions. miRNA isolated from cells, tissues, and sera were obtained using the miRNA Isolation Kit and mirVana PARIS Kit (Applied Biosystems, Foster City, CA, USA), according to the manufacturer's protocol. The specific sources of miRNAs were: 10^6 ^to 10^7 ^cells (described above), 100 mg of tissue from each of 70 NSCLC patients and matched adjacent noncancerous tissues, and circulating miRNAs from 600 μL of serum from each of 70 NSCLC patients and 44 normal volunteers. The RNA concentration was measured by a NanoDrop ND-1000 spectrophotometer (nm readings: A260/280 > 2.0, A260/230 > 1.8; NanoDrop Technologies).

### MicroRNA microarray and data analysis

Three to five micrograms of total RNA were labeled by ligating the fluorescent RNA-linker 5'-cytidine bisphosphate-cyanine 3 (pCp-Cy3) to the 3' end of miRNAs. Slides were then incubated with the labeled RNA and washed. Five hundred and nine miRNAs passed the initial screening criteria of the normalized median fluorescence signal. The images were analyzed using SpotReader software (Niles Scientific, Portola Valley, CA). The significance of differences in expression levels was assessed by a two-sided paired *t*-test within the significance analysis of microarrays (SAM). *P *< 0.05 indicated a significant difference.

### RT-qPCR of miRNA derived from lung cancer cell lines and lung primary tissues

The amounts of the miRNAs in lung tumors, analyzed by microarray, were quantified by RT-qPCR using TaqMan MicroRNA Assay Kits (Applied Biosystems, USA). Briefly, the reverse transcription (RT) reaction was carried out with a TaqMan MicroRNA Reverse Transcription Kit (Applied Biosystems) according to the instruction of the protocol. One to ten nanograms of total RNA per 15 μL RT reaction were processed at 16°C for 30 min, 42°C for 30 min, and 85°C for 5 min. Following the RT, quantitative real-time PCR was performed in an ABI 7500 Real-Time PCR system (Applied Biosystems) at 95°C for 10 min, followed by 40 cycles of 95°C for 15 s and 60°C for one minute.

The cycle threshold (Ct) values were calculated with SDS 2.0.1 software (Applied Biosystems). The average expression levels of miRNAs in tissues and sera were normalized with U6 small nuclear RNA (snRNA) and U48 snRNA using the 2^-ΔΔCt ^method [23]. The mean Ct value in three candidate miRNAs was calculated, excluding outliers (i.e., replicates with a Ct differing by > one cycle from the median). If Ct_U6, ave _and Ct_U48, ave _were not within 20 and 32 cycles, the assay was repeated. Samples with low U6 or U48 snRNA levels were not included in this study.

### Statistical analyses

The statistical analyses were performed with Graphpad Prism 5.0 statistical software. The data were examined according to the degree of homogeneity. The paired *t*-test, unpaired *t*-test, or Mann-Whitney *U*-test was used to analyze the correlation between the miRNA expression levels and clinical-pathological features of the patients. The paired sample *t*-test was used to compare the differences in miRNA expression between lung tissues and sera. All data were expressed as the mean ± standard error of the mean (SEM). Binary logistic regression was used to assess associations between the miR-183 family and the clinical-pathological features of the patients. Survival analysis was performed with the Kaplan-Meier method, and the log-rank test was used to compare survival times between groups and was adjusted according to age and tumor stages. The Cox hazard regression model was used to analyze the miRNAs as risk factors for lung cancer. A probability (*P*)-value < 0.05 was considered statistically significant.

## Results

### Expression of the miR-183 family of miRNAs in lung tissues, sera, and cell lines

MicroRNA arrays were performed from 6 lung cancer and 6 matched adjacent normal lung tissues that contained 723 human miRNA probes (from version 10.0 of miRBase sequence database, http://www.mirbase.org/). A total of 94 miRNAs were differentially expressed between lung carcinoma and the normal counterpart. Among those, we selected for further study 3 cluster miRNAs, namely miR-96, miR-182, and miR-183, based on the magnitude of fold changes (10.77-, 10.75-, and 6.62-fold, respectively) and probability values (*P *= 0.00127, 0.00209 and 0.00190), as shown in our previous study [24].

**Figure 1:**
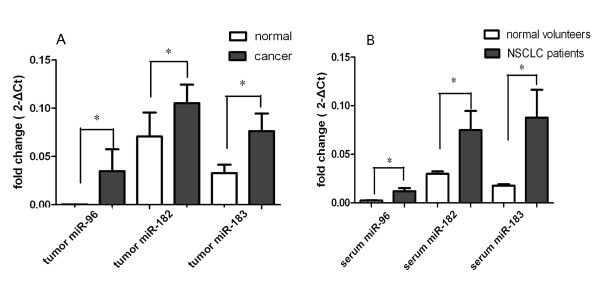
**RT-qPCR analysis of miRNAs in lung carcinoma and matched noncancerous tissues**. (A) Paired sample *t*-test was used to compare the differential expressions of miR-96, miR-182, and miR-183 in 70 lung carcinoma and matched noncancerous tissues. The *P*-values for miR-96, miR-182, and miR-183 were *P <*0.0001 for each. (B) Unpaired sample *t*-test was used to compare the differential expressions of miR-96, miR-182, and miR-183 in sera from 70 lung cancer patients and 44 healthy controls. The *P*-values for miR-96, miR-182, and miR-183 were *P <*0.0001, *= *0.0130, and *= *0.0086, respectively. *Indicates a significant difference between groups (*P *< 0.05).

We validated the three miRNAs in 70 pairs of lung carcinoma and the corresponding noncancerous lung tissues by RT-qPCR. We showed that, compared with the adjacent normal lung tissues, the levels of the mature forms of miR-96, miR-182, and miR-183 were significantly increased in lung cancer tissues (*P <*0.0001 for each; Figure 1A). The highly expressed (> 2-fold changes) miR-96, miR-182 and miR-183 were observed in 42 (60.0%), 54 (77.1%) and 48 (68.6%) of the 70 samples, respectively.

**Figure 2:**
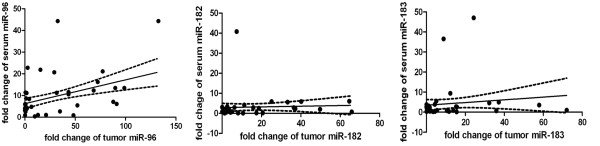
**Correlation between the expressions of miR-96, miR-182, and miR-183 in tissues vs. sera from lung cancer patients**. Pearson's correlation between the expression of miR-96, miR-182, and miR-183 in NSCLC tissues and sera are shown. The correlation rate of miR-96 was 0.419, *P = *0.0003; miR-182 was 0.0464, *P = *0.703; and miR-183 was 0.118, *P = *0.332.

Compared to the sera of normal volunteers, the expression levels of miR-96, miR-182, and miR-183 in the sera of NSCLC patients were significantly higher in 62 (88.6%), 38 (54.3%), and 32 (45.7%) of the 70 samples (*P <*0.0001, *= *0.0130, and *= *0.0086, respectively; Figure 1B). A correlation was also found between the serum and tumor levels of miR-96 (*Pearson r = *0.419, *P = *0.0003), but not for miR-182 (*Pearson r *= 0.0464, *P = *0.703) or miR-183 (*Pearson r = *0.118, *P = *0.332). This suggested that serum levels of miR-96, but not miR-182 and miR-183, might correspond to the levels of tumor miR-96 (Figure 2).

**Figure 3:**
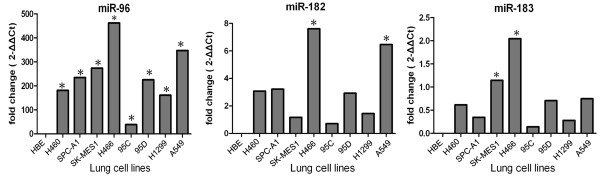
**RT-qPCR analysis of lung cancer cell lines**. Total RNA was isolated from several different lung cancer cells lines by the Trizol method (Invitrogen) according to the manufacturer's instructions, and qRT-PCR was performed using TaqMan MicroRNA Assay Kits (Applied Biosystems). The average expression levels of miR-96, miR-182, and miR-183 in tissues and sera were normalized using the 2-^ΔΔCt ^method relative to the average of U6 snRNA and U48 snRNA. The average Ct value of triplicates in three candidate miRNAs was calculated. *Indicates a significant difference compared to HBE cells (*P *< 0.05).

Finally, we tested levels of miRNAs in different types of lung cancer vs. normal cell lines. The results from RT-qPCR were expressed as fold changes in each miRNA in lung cancer cells relative to normal cells. As shown in Figure 3, the expression levels of miR-96 were higher in 8 lung cancer cell lines. High levels of miR-182 were found in H466 and A549 cells, and high levels of miR-183 in H466 and SK-MES1. The highest expression levels for the three miRNAs were found in NCI-H466 cells. In addition, the expression levels of all three were higher in A549 and 95D cells than in H1299 and 95C cells.

### Correlation between miR-183 family and clinical-pathological features of NSCLC

**Figure 4:**
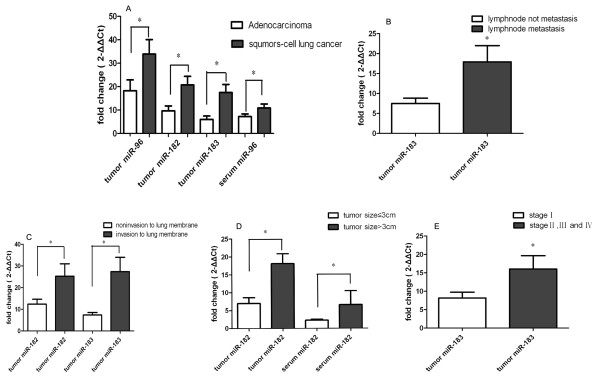
**Correlation of expression of miR-183 family with clinical-pathological features of lung cancer**. The Mann-Whitney U-test was used to examine the correlation between miR-183 family levels and clinical-pathological features. The results showed that (A) Tumor miR-96, miR-182, and miR-183, and serum miR-96 expression were higher in squamous-cell lung carcinoma than in adenocarcinoma (*P = *0.0216, 0.0190, 0.0042, and 0.0310, respectively). (B) Tumor miR-183 expression was higher in NSCLC patients with lymph node metastasis than without lymph node metastasis (*P = *0.0086). (C) Tumor miR-182 and miR-183 expressions were higher in NSCLC patients with tumor invasion to lung membrane than without invasion to lung membrane (*P = *0.0222 and 0.0140, respectively). (D) Tumor and serum miR-182 levels were higher in NSCLC patients with tumor size > 3 cm than those with tumor size < 3 cm (*P = *0.0464 and 0.0351, respectively). (E) Tumor miR-183 expression was higher in stages II, III, and IV NSCLC samples than in stage I. Differences were significant after the Mann Whitney *U*-test. *Indicates a significant difference between groups (*P *< 0.05).

We also analyzed with the Mann-Whitney *U*-test the correlation between miRNAs and clinical-pathological features of NSCLC (including age, gender, smoking history, histological classification, lymph node metastasis, differentiation, and clinical-pathological stage) to understand better the potential role of these miRNAs in NSCLC development and progression. Our results showed that overexpression of miR-183 in tumors was strongly associated with lymph node metastasis, invasion of the lung membrane, and advanced clinical stage of NSCLC (*P = *0.0086, 0.0222 and 0.0478, respectively). Overexpression of miR-182 in tumors was positively related to invasion of the lung membrane and tumor size > 3 cm (*P *= 0.0140 and 0.0464, respectively). In sera, miR-182 overexpression was also positively related to invasion of the lung membrane and tumor size > 3 cm (P = 0.0222 and 0.0351, respectively). Compared to lung adenocarcinoma, higher expression of miR-96, miR-182, and miR-183 in tumors, and miR-96 in sera, were found in squamous cell lung carcinoma (*P = *0.0216, 0.0190, 0.0042, and 0.0310, respectively; Figure 4).

We also analyzed the association between levels of the miR-183 family and clinical-pathological features with a binary logistic regression model. Results showed that patients over 60 years old, or with squamous-cell lung carcinoma, had higher levels of tumor miR-96. Patients with squamous-cell lung carcinoma or tumor invasive to lung membrane had higher levels of tumor miR-183. Patients with poor differentiation had higher levels of miR-96, miR-182 and miR-183 in sera (Table 2).

**Table 2:** Binary logistic regression analysis for an association between the expression levels of the miR-183 family and clinical-pathological features of patients

	subset	Exp (B) (95% confidence interval)	*P*-value
Tumor miR-96	High, > 6.45/low, < 6.45		
Age	< 60/≥60	2.857 (1.080-7.559)	0.034*
Histological classification	adenocarcinoma/SCC	3.667 (1.366-9.842)	0.010*
**Tumor miR-183**	**High, > 4.44/low, < 4.44**		
**Histological classification**	**adenocarcinoma/SCC**	**3.667 (1.366-9.842)**	0.010*
**Invasion to lung membrane**	**Negative/positive**	**3.750 (1.072-13.121)**	0.039*
Serum miR-96	High, > 5.98/low, < 5.98		
Differentiation	poor/Mod-well	0.321 (0.114-0.905)	0.032*
**Serum miR-182**	**High, > 2.08/low, < 2.08**		
**Differentiation**	**poor/Mod-well**	**0.178 (0.059-0.537)**	0.002*
Serum miR-183	High, > 1.84/low, < 1.84		
Differentiation	poor/Mod-well	0.321 (0.114-0.905)	0.032*

Binary logistic regression analysis. *Indicates a significant difference (*P <*0.05).

### Correlation between expression of miRNAs of the miR-183 family and overall survival of NSCLC patients

**Figure 5:**
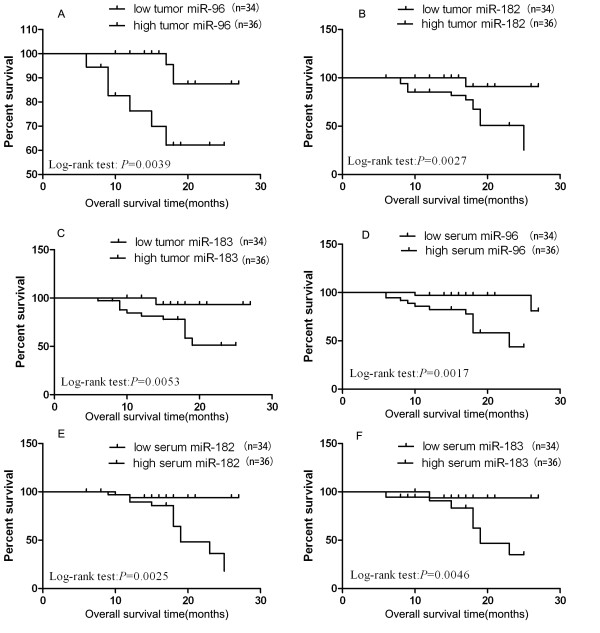
**Kaplan-Meier survival curves for NSCLC patients plotted for tumor and serum miR-96, miR-182, and miR-183**. Kaplan-Meier survival curves for NSCLC patients based on the median level of fold change. The *P*-value was calculated using the log-rank test between patients with high- and low-fold changes. Overall survival of patients with high vs. low miR-183 family expression levels are shown. *P *< 0.05 indicates a significant difference between groups.

Next, we investigated whether the expressions of miR-96, miR-182, and miR-183 were correlated with overall survival of NSCLC patients. Specifically, we used the Kaplan-Meier method, log-rank test and univariate Cox hazard regression model to analyze the correlation between overall survival of NSCLC patients and the expressions of miR-96, miR-182, and miR-183 in tumors (medians of 2^-ΔΔCt ^values: 6.45, 6.25, and 4.44, respectively) and in sera (medians 5.98, 2.08, and 1.84). The results showed that high expressions of miR-96, miR-182 and miR-183 in tumors and sera were independently correlated with shorter overall survival of NSCLC patients (log-rank test: *P = *0.0039, 0.0027, 0.0053, respectively in tumors, and *P = *0.0017, 0.0025, and 0.0046 in sera; Figure 5).

We also examined if the expressions of miR-96, miR-182, and miR-183 in tumors and sera were correlated with clinical-pathological features of the patients, with hazard ratios (adjusted for sex, age and tumor stage) of 9.637, 7.163, 8.616, *P *= 0.005, 0.010, and 0.005 respectively in tumors and with hazard ratios (adjusted for sex, age and tumor stage) of 5.512, 5.327, 5.972 yielded *P *= 0.027, 0.030, and 0.022 respectively (Table 3) in sera. This result suggested that miR-96, miR-182 and miR-183 in tumor and sera independently contributed to the overall survival of lung cancer patients

**Table 3:** Univariate Cox hazard regression analysis for prognostic factors

	subset	Adjusted Hazard ratio (95% confidence interval)	*P-*value
Gender	male/female	0.031 (0.000-5.569)	0.190
**Age**	**< 60/≥60**	**1.630 (0.564-4.716)**	**0.367**
Smoking	nonsmoker/current smoker	1.388 (0.306-6.297)	0.671
**Tumor size**	**0-3 cm/> 3 cm**	**0.766 (0.239-2.455)**	**0.654**
Histological classification	adenocarcinoma/SCC	2.739 (0.781-9.604)	0.115
**Invasion to lung membrane**	**Negative/positive**	**1.000 (0.221-4.534)**	**1.000**
Differentiation	Mod-well/poor	0.757 (0.182-3.141)	0.701
**Lymph node**	**Negative/positive**	**1.279 (0.425-3.850)**	**0.661**
Stage classification	stage I/stage II, III, IV	1.001 (0.334-2.996)	0.999
**Tumor miR-96**	**High, > 6.45/low, < 6.45**	**9.637 (1.963-47.315)**	0.005 *
Tumor miR-182	High, > 6.25/low, < 6.25	7.163 (1.597-32.138)	0.010 *
**Tumor miR-183**	**High, > 4.44/low, < 4.44**	**8.616 (1.918-38.705)**	0.005*
Serum miR-96	High, > 5.98/low, < 5.98	5.512 (1.219-24.915)	0.027 *
**Serum miR-182**	**High, > 2.08/low, < 2.08**	**5.327 (1.181-24.030)**	0.030 *
Serum miR-183	High, > 1.84/low, < 1.84	5.972 (1.289-27.658)	0.022 *

Univariate cox hazard regression analysis. *indicates a significant difference (*P <*0.05).

## Discussion

Lung carcinoma is one of the most common malignancies in the world, and is the leading cause of cancer death in men and women in the United States with a dismal five-year survival rate (< 15%) [1]. Although recent advances have been made in improving diagnosis and treatment strategies, the prognosis of lung cancer patients remains unchanged and early detection is critical in improving survival duration. Studies have shown that miRNAs play an important role in tumorigenesis and they have been considered potential biomarkers for early diagnosis and prognosis in a wide variety of human cancers [6,14,25,26]. More importantly, circulating miRNAs might act as noninvasive blood-based biomarkers for cancer diagnosis [14,27]. One recent report demonstrated that miRNAs in tissues and plasma could have a critical role as molecular predictors of lung cancer development and management outcome [28].

The upregulation of members of the miR-183 family (miR-96, miR-182, miR-183) has been reported in several types of cancers [15-20]. Consistent with our results, members of the miR-183 family were elevated in lung cancer, revealed by novel rank-based statistical methods using microarrays [21,22,29]. miR-96, a known onco-miRNA, targets the tumor suppressor gene forkhead box O3 (*FOXO3*; a transcription factor that plays important roles in tumorigenesis) by binding to the 3^'^-UTR of *FOXO3 *mRNA and thereby mediating protection against apoptosis and promoting cell survival [16,30]. Studies have also reported that miR-27a, miR-96, miR-182, and miR-183 coordinately regulated the expression of forkhead box O1 (*FOXO1*) by directly targeting the mRNA *FOXO1 *3'-UTR [31,32]. In addition, miR-183 functioned as an onco-miRNA, shown to target the tumor suppressor transcription factor early growth response 1 (*EGR1*), and phosphatase and tensin homolog (*PTEN*) to promote tumor cell migration [19].

Consistent with other reports [6,19,32], we showed that compared to adjacent non-cancerous lung tissues and normal volunteers, members of the miR-183 family were highly expressed in lung cancer primary tissues and sera. Moreover, we found that the expression levels of miR-96, miR-182, and miR-183 were higher in A549 and 95D cells than in H1299 and 95C cells, respectively. This implies that the miR-183 family might participate in tumor metastasis [19,30,33,34]. Consistent with this, other studies demonstrated that overexpressed miR-96 and miR-182 in melanoma cell lines and breast cancer cell lines resulted in enhanced oncogenic properties as well as invasion and metastasis *in vitro *[30,33]. Sarver *et al*. [19] showed that a reduction in miR-183 led to a decrease in migration in colon cancer and synovial sarcoma cell lines. Abraham *et al*. [34] reported that overexpression of miR-183 in medullary thyroid cancer predicted lymph node metastasis.

We note that opposite results have also been reported. According to one study, expression of miR-96 decreased cell invasion and migration in pancreatic cancer [35]. Another report demonstrated that miR-182 suppressed cell proliferation of lung cancer cell line A549 *in vitro *and tumor growth *in vivo *through its interference with the target gene cortactin (*CTTN*) by epigenetic modification [36]. Others found that overexpression of miR-183 inhibited migration and invasion of lung cancer and breast cancer cells, by targeting the protein ezrin [22,37]. These discrepancies might be due to different cancer cell lines used, and suggest that miRNA might have distinct functions depending upon cell type [38].

Our results demonstrate an association between the expressions of miR-183 and miR-182 and metastasis, invasion, advanced clinical stage, and tumor sizes of NSCLC. Together with other reports, they imply that miR-182 and miR-183 might play a role in lung cancer invasion and metastasis, and progression of NSCLC [28,30]. Moreover, in our study patients with high tumor or serum levels of miR-96, miR-182, and miR-183 had shorter overall post-operative survival times. The hazard ratios adjusted for gender, age and tumor stage indicated that these miRNAs could be used independently for evaluating the prognoses of NSCLC patients.

In addition, our results demonstrated that serum miRNAs might be used as potential biomarkers for diagnosis and prognosis of lung cancers. Under lung cancer condition, miRNAs entered the circulation mainly from tumor cells [27]. One study showed that miRNAs in tissue specimens were not detected by Solexa sequencing in sera [39]. By comparing the expressions of miR-96, miR-182 and miR-183 in tumors and sera, our results suggest that the levels of serum and tumor miR-96 were correlated, while those of miR-182 and miR-183 were not. These results are consistent with reports from Mitchell [27] and Hu [39]. The actual role and functions of these miRNAs need to be further investigated.

## Conclusions

Our results show that members of the miR-183 family, found in tumors and sera, may play a role in the development of NSCLC and have potential as biomarkers in the diagnosis and prognosis of lung cancer. We further demonstrate a positive correlation between expression levels of miR-96 in tumors and sera from NSCLC patients. Further study needs to be done to define the true value of combined expression profiles of the miR-183 family in tumors and sera in the diagnosis and prognosis of lung cancer.

## List of abbreviations

**Ct: **cycle threshold; ***CTTN*: **cortactin; **DMEM: **Dulbecco's modified Eagle's medium; ***EGR1*: **early growth response 1; ***FOXO3 ***forkhead box O3; ***FOXO1*: **forkhead box O1; **miRNA: **microRNA; **NCCN: **National Comprehensive Cancer Network; **NSCLC: **non-small cell lung cancer; **pCp-Cy3: **5'-cytidine bisphosphate-cyanine 3; ***PTEN*: **phosphatase and tensin homolog; **RT: **reverse transcription; **qRT-PCR: **quantitative real-time reverse transcription polymerase chain reaction; **SAM: **significance analysis of microarrays; **SEM: **standard error of the mean; **snRNA: **small nuclear RNA.

## Competing interests

The authors declare that they have no competing interests.

## Authors' contributions

WYZ, XGL, and YKZ were involved in the conception and design of the study. WYZ, JYH, DDC, and YYH were involved in the provision of study material and patients. WYZ and XGL performed the data analysis and interpretation. WYZ wrote the manuscript. YKZ approved the final version. All authors read and approved the final manuscript.

## Pre-publication history

The pre-publication history for this paper can be accessed here:

http://www.biomedcentral.com/1471-2407/11/393/prepub

## References

[B1] Jemal A Siegel R Ward E Hao Y Xu J Thun MJ Cancer statistics, 2009 CA-Cancer J Clin 2009 59 225 249 10.3322/caac.20006 19474385

[B2] Jay C Nemunaitis J Chen P Fulgham P Tong AW miRNA profiling for diagnosis and prognosis of human cancer DNA Cell Biol 2007 26 293 300 10.1089/dna.2006.0554 17504025

[B3] Cummins J Velculescu V Implications of micro-RNA profiling for cancer diagnosis Oncogene 2006 25 6220 6227 10.1038/sj.onc.1209914 17028602

[B4] Yu SL Chen HY Chang GC Chen CY Chen HW Singh S Cheng CL Yu CJ Lee YC Chen HS MicroRNA signature predicts survival and relapse in lung cancer Cancer Cell 2008 13 48 57 10.1016/j.ccr.2007.12.008 18167339

[B5] Calin GA Croce CM MicroRNA-cancer connection: the beginning of a new tale Cancer Res 2006 66 7390 7394 10.1158/0008-5472.CAN-06-0800 16885332

[B6] Volinia S Calin GA Liu CG Ambs S Cimmino A Petrocca F Visone R Iorio M Roldo C Ferracin M A microRNA expression signature of human solid tumors defines cancer gene targets Proc Natl Acad Sci 2006 103 2257 2261 10.1073/pnas.0510565103 16461460 PMC1413718

[B7] Rosell R Wei J Taron M Circulating microRNA signatures of tumor-derived exosomes for early diagnosis of non-small cell lung cancer Clin Lung Cancer 2009 10 8 9 10.3816/CLC.2009.n.001 19289365

[B8] Ventura A Jacks T MicroRNAs and cancer: short RNAs go a long way Cell 2009 136 586 591 10.1016/j.cell.2009.02.005 19239879 PMC3910108

[B9] Yanaihara N Caplen N Bowman E Seike M Kumamoto K Yi M Stephens RM Okamoto A Yokota J Tanaka T Unique microRNA molecular profiles in lung cancer diagnosis and prognosis Cancer cell 2006 9 189 198 10.1016/j.ccr.2006.01.025 16530703

[B10] Raponi M Dossey L Jatkoe T Wu X Chen G Fan H Beer DG MicroRNA classifiers for predicting prognosis of squamous cell lung cancer Cancer Res 2009 69 5776 5783 10.1158/0008-5472.CAN-09-0587 19584273

[B11] Galluzzi L Morselli E Vitale I Kepp O Senovilla L Criollo A Servant N Paccard C Hupe P Robert T MiR-181a and miR-630 regulate cisplatin-induced cancer cell death Cancer Res 2010 70 1793 1803 10.1158/0008-5472.CAN-09-3112 20145152

[B12] Zhao H Shen J Medico L Wang D Ambrosone CB Liu S Creighton C A pilot study of circulating miRNAs as potential biomarkers of early stage breast cancer PLoS One 2010 5 876 887 10.1371/journal.pone.0013735 PMC2966402 21060830

[B13] Resnick KE Alder H Hagan JP Richardson DL Croce CM Cohn DE The detection of differentially expressed microRNAs from the serum of ovarian cancer patients using a novel real-time PCR platform Gynecol oncol 2009 112 55 59 10.1016/j.ygyno.2008.08.036 18954897

[B14] Chen X Ba Y Ma L Cai X Yin Y Wang K Guo J Zhang Y Chen J Guo X Characterization of microRNAs in serum: a novel class of biomarkers for diagnosis of cancer and other diseases Cell Res 2008 18 997 1006 10.1038/cr.2008.282 18766170

[B15] Gokhale A Kunder R Goel A Sarin R Moiyadi A Shenoy A Mamidipally C Noronha S Kannan S Shirsat N Distinctive microRNA signature of medulloblastomas associated with the WNT signaling pathway J Cancer Res Ther 2010 6 521 529 10.4103/0973-1482.77072 21358093

[B16] Lin P Yu S Yang P MicroRNA in lung cancer Br J cancer 2010 103 1144 1148 10.1038/sj.bjc.6605901 20859290 PMC2967070

[B17] Schaefer A Jung M Mollenkopf HJ Wagner I Stephan C Jentzmik F Miller K Lein M Kristiansen G Jung K Diagnostic and prognostic implications of microRNA profiling in prostate carcinoma Int J Cancer 2010 126 1166 1176 19676045 10.1002/ijc.24827

[B18] Pineau P Volinia S McJunkin K Marchio A Battiston C Terris B Mazzaferro V Lowe SW Croce CM Dejean A miR-221 overexpression contributes to liver tumorigenesis Proc Natl Acad Sci 2010 107 264 269 10.1073/pnas.0907904107 20018759 PMC2806773

[B19] Sarver AL Li L Subramanian S MicroRNA miR-183 functions as an oncogene by targeting the transcription factor EGR1 and promoting tumor cell migration Cancer Res 2010 70 9570 9580 10.1158/0008-5472.CAN-10-2074 21118966

[B20] Bandres E Cubedo E Agirre X Malumbres R Zarate R Ramirez N Abajo A Navarro A Moreno I Monzo M Identification by Real-time PCR of 13 mature microRNAs differentially expressed in colorectal cancer and non-tumoral tissues Mol Cancer 2006 5 29 16854228 10.1186/1476-4598-5-29 PMC1550420

[B21] Navon R Wang H Steinfeld I Tsalenko A Ben-Dor A Yakhini Z Novel rank-based statistical methods reveal microRNAs with differential expression in multiple cancer types PloS one 2009 4 e8003 10.1371/journal.pone.0008003 19946373 PMC2777376

[B22] Wang J Chen J Chang P LeBlanc A Li D Abbruzzesse JL Frazier ML Killary AM Sen S MicroRNAs in plasma of pancreatic ductal adenocarcinoma patients as novel blood-based biomarkers of disease Cancer Prev Res 2009 2 807 10.1158/1940-6207.CAPR-09-0094 PMC5859193 19723895

[B23] Livak KJ Schmittgen TD Analysis of relative gene expression data using real-time quantitative PCR and the 2 (-Delta Delta C (T)) method Methods 2001 25 402 408 10.1006/meth.2001.1262 11846609

[B24] Liu X Zhu W Huang Y Ma L Zhou S Wang Y Zeng F Zhou J Zhang Y High expression of serum miR-21 and tumor miR-200c associated with poor prognosis in patients with lung cancer Med Oncol 2011 in press 10.1007/s12032-011-9923-y 21516486

[B25] Lu J Getz G Miska EA Alvarez-Saavedra E Lamb J Peck D Sweet-Cordero A Ebert BL Mak RH Ferrando AA MicroRNA expression profiles classify human cancers Nature 2005 435 834 838 10.1038/nature03702 15944708

[B26] Asaga S Kuo C Nguyen T Terpenning M Giuliano AE Hoon DSB Direct serum assay for microRNA-21 concentrations in early and advanced breast cancer Clin Chem 2011 57 84 91 10.1373/clinchem.2010.151845 21036945

[B27] Mitchell PS Parkin RK Kroh EM Fritz BR Wyman SK Pogosova-Agadjanyan EL Peterson A Noteboom J O'Briant KC Allen A Circulating microRNAs as stable blood-based markers for cancer detection Proc Natl Acad Sci 2008 105 10513 10518 10.1073/pnas.0804549105 18663219 PMC2492472

[B28] Boeri M Verri C Conte D Roz L Modena P Facchinetti F Calabr E Croce CM Pastorino U Sozzi G MicroRNA signatures in tissues and plasma predict development and prognosis of computed tomography detected lung cancer Proc Natl Acad Sci 2011 108 3713 3718 10.1073/pnas.1100048108 21300873 PMC3048155

[B29] Barshack I Lithwick-Yanai G Afek A Rosenblatt K Tabibian-Keissar H Zepeniuk M Cohen L Dan H Zion O Strenov Y MicroRNA expression differentiates between primary lung tumors and metastases to the lung Pathol Res Prac 2010 206 578 584 10.1016/j.prp.2010.03.005 20418022

[B30] Segura MF Hanniford D Menendez S Reavie L Zou X Alvarez-Diaz S Zakrzewski J Blochin E Rose A Bogunovic D Aberrant miR-182 expression promotes melanoma metastasis by repressing FOXO3 and microphthalmia-associated transcription factor Proc Natl Acad Sci 2009 106 1814 1819 10.1073/pnas.0808263106 19188590 PMC2634798

[B31] Guttilla IK White BA Coordinate regulation of FOXO1 by miR-27a, miR-96, and miR-182 in breast cancer cells J Biol Chem 2009 284 23204 23216 10.1074/jbc.M109.031427 19574223 PMC2749094

[B32] Myatt SS Wang J Monteiro LJ Christian M Ho KK Fusi L Dina RE Brosens JJ Ghaem-Maghami S Lam EWF Definition of microRNAs that repress expression of the tumor suppressor gene FOXO1 in endometrial cancer Cancer Res 2010 70 367 377 10.1158/0008-5472.CAN-09-1891 20028871 PMC2880714

[B33] Li J Fu H Xu C Tie Y Xing R Zhu J Qin Y Sun Z Zheng X miR-183 inhibits TGF-β1-induced apoptosis by downregulation of PDCD 4 expression in human hepatocellular carcinoma cells BMC cancer 2010 10 354 10.1186/1471-2407-10-354 20602797 PMC2909210

[B34] Abraham D Jackson NE Gundara JS Zhao J Gill AJ Delbridge L Robinson B Sidhu S MicroRNA profiling of sporadic and hereditary medullary thyroid cancer identifies predictors of nodal metastasis, prognosis and potential therapeutic targets Clin Cancer Res 2011 17 4772 4781 10.1158/1078-0432.CCR-11-0242 21622722

[B35] Yu S Lu Z Liu C Meng Y Ma Y Zhao W Liu J Yu J Chen J miRNA-96 suppresses KRAS and functions as a tumor suppressor gene in pancreatic cancer Cancer Res 2010 70 6015 6025 10.1158/0008-5472.CAN-09-4531 20610624

[B36] Zhang L Liu T Huang Y Liu J microRNA-182 inhibits the proliferation and invasion of human lung adenocarcinoma cells through its effect on human cortical actin-associated protein Int J Mol Med 2011 28 381 383 21503569 10.3892/ijmm.2011.679

[B37] Lowery AJ Miller N Dwyer RM Kerin MJ Dysregulated miR-183 inhibits migration in breast cancer cells BMC cancer 2010 10 502 10.1186/1471-2407-10-502 20858276 PMC2955037

[B38] Hyun S Lee JH Jin H Nam JW Namkoong B Lee G Chung J Kim VN Conserved MicroRNA miR-8/miR-200 and its target USH/FOG2 control growth by regulating PI3K Cell 2009 139 1096 1108 10.1016/j.cell.2009.11.020 20005803

[B39] Hu Z Chen X Zhao Y Tian T Jin G Shu Y Chen Y Xu L Zen K Zhang C Serum microRNA signatures identified in a genome-wide serum microRNA expression profiling predict survival of non-small cell lung cancer J Clin Oncol 2010 28 1721 1726 10.1200/JCO.2009.24.9342 20194856

